# Intensively Reared Nelore Steers with Levels of Concentrate and Protein Sources during the Dry Season

**DOI:** 10.3390/ani14121787

**Published:** 2024-06-14

**Authors:** Artur C. de Faria, Dheyme C. Bolson, Douglas dos S. Pina, Thiago A. Prado, Adriano N. Roecker, Carla S. Chaves, Dalton H. Pereira

**Affiliations:** 1 Grupo de Estudos em Pecuária Integrada-GEPI, Universidade Federal de Mato Grosso, Sinop 78550-728, Mato Grosso, Brazil; arturcarmanini1996@gmail.com (A.C.d.F.); adrianor96@gmail.com (A.N.R.); carlazootecnia@gmail.com (C.S.C.); 2 Departamento de Pesquisa e Desenvolvimento da Fortuna Nutrição Animal, MT-320 km 198 Zona Rural, Nova Canaã do Norte 78515-000, Mato Grosso, Brazilthiago@nafortuna.com.br (T.A.P.); 3 Animal Science Department, Universidade Federal da Bahia, Salvador 40170-115, Bahia, Brazil; douglaspinaufba@gmail.com

**Keywords:** beef cattle, DDGS, intensification, *Megathyrsus maximus*, blood parameters, intensive rearing

## Abstract

**Simple Summary:**

The dry season is the most challenging season for beef cattle grazing. Intensification during the dry season ensures that animal performance is maintained and allows for increases in productivity per area. Thus, the use of concentrated supplementation not only ensures the adjustment of limiting nutrients in the pasture but also allows for the manipulation of the individual and area gain curve. The effects of nutritional strategies associated with supplementation levels and protein sources on the performance, blood parameters, and forage composition of Nelore animals in the rearing phase during the dry period of the year were evaluated. Two protein sources (PS) (soybean meal—SBM and dry distillers grain with soluble—DDGS) and two levels of concentrate (LC) (higher and lower) were tested in the Mombaça grass pastures. Higher levels of concentrate ensure greater productivity for beef cattle grazing. The highest LC increased the total weight gain of beef cattle grazing by 50.5%, while a lower LC increased the leaf/stem ratio (L/S) and crude protein (CP) content of Mombasa grass.

**Abstract:**

The objective of this study was to evaluate the effect of different nutritional strategies on the intensification of beef cattle farming on pastures during the dry period of the year. Eighty male cattle (testers) were randomly allocated to 16 paddocks formed with Mombaça grass (*Megathyrsus maximus*), totaling five animals (testers) per paddock. The strategies consisted of two LCs [10 and 16.7 g·kg^−1^ body weight (BW)] and two PSs with DDGS and SBM in a completely randomized design with a 2 × 2 factorial arrangement. The chemical, structural, and productive characteristics of the forage were evaluated, as well as the performance, productivity, and serum parameters of the supplemented animals. The forage presented a greater L:C (*p* = 0.033) and CP content (*p* = 0.007) when the lowest LC was used. Animals that received the highest LC had the highest supplement intake (*p* < 0.001) and the lowest pasture intake (*p* < 0.001). The nutritional strategy with an LC of 16.7 g·kg^−1^ of body weight (BW) resulted in a greater increase in total BW, i.e., 200 kg·BW ha^−1^ more. Therefore, higher levels of concentrate ensure greater productivity for beef cattle grazing, and DDGS can replace SBM in supplements used in the intensive raising of beef cattle on pasture without compromising the performance and productivity of the animals.

## 1. Introduction

In central Brazil, the seasons are distinct, with dry winters and rainy summers, differentiated mainly by rainfall. This distinction gives special characteristics to grazed forages in terms of quantity and quality. However, pasture as an exclusive food source is not capable of meeting all the nutritional requirements for animal gain [1,2], especially during the dry period of the year, a time characterized by a reduction in the supply of forage and the quality of the material available [3]. Thus, the use of concentrated supplementation, in addition to ensuring the adjustment of limiting nutrients in the pasture [4], allows for the individual and area gain curve to be manipulated [5], resulting in lower greenhouse gas emissions [6,7]. According to these authors, diets containing high levels of starch (concentrates) emit less CH_4_ than those composed principally of structural carbohydrates (grasses).

When considering supplementation for pastured animals, the protein ingredients represent the most expensive input to be used for supplementation. Soybean meal is the main protein ingredient in the ruminant diet; however, it is an input that is used to feed other livestock, which contributes to the increase in its cost. In this sense, distillers grain (DDGS) can be used as an alternative ingredient to formulate supplements for beef cattle [6]. The DDGS byproducts are also competitively priced, especially in producing regions such as Mato-Grosso, Brazil [8].

During the rearing phase, concentrated supplementation is an intensification strategy that accelerates the growth curve of the animals by meeting the protein requirement, which would meet the demand for the deposition of muscle tissue, the main tissue deposited in this phase [9]. Furthermore, intensification through the use of high levels of supplements in the feed of grazing ruminants is one of the main production strategies in competitive livestock farming. However, it was observed that the maximum level of supplementation carried out during rearing is 6 g·kg^−1^ BW [10], leaving a gap as to what the response of the production system would be to levels above these.

Supplementation during the dry season allows for the maintenance and/or increase in average daily gain (ADG) and, in certain situations, an increase in production per area [11]. However, there are little data in the national and global literature on high levels (above 7 g·kg^−1^ BW) of supplementation during the rearing phase of beef cattle, especially when supplemented with alternative protein sources. Thus, we hypothesize that greater quantities of concentrate can promote greater productivity of steers during drought, with the possibility of replacing SBM with DDGS without affecting individual performance. Therefore, the objective of this study was to evaluate the effect of nutritional strategies combining supplementation levels and protein sources on the performance, blood parameters, and forage composition of Nelore animals in the rearing phase during the dry period of the year.

## 2. Materials and Methods

### 2.1. Ethical Approval

The protocol used in this study was under the Brazilian College of Animal Experimentation guidelines (COBEA—Brazilian College of Animal Experimentation) and was approved by the National Organization for Animal Experimentation Control (CONCEA—National Council for the Control of Animal Experimentation) of the UFMT—Araguaia campus (protocol number 23108.042666/2020-94).

### 2.2. Experimental Area

**Figure 1:**
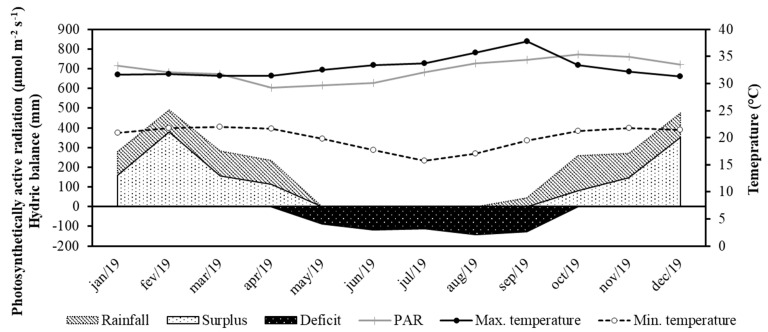
Deficit and excess water in the soil, accumulated precipitation, and minimum and maximum air temperatures from January 2019 to December 2019.

The study was conducted at the experimental center of the Fortuna Nutrição Animal Company at Fazenda Gamada, located in the municipality of Nova Canaã do Norte, state of Mato-Grosso, Brazil (10°24′35″ W, 55°43′35″ S, altitude 288 m). The experimental area included 40 ha of Mombasa grass (*Megathyrsus maximus*) pasture, which was subdivided into 16 paddocks of 2.5 ha each. Each paddock was equipped with 500 liters (L) of drinking fountains and a 6 m linear trough with free access. The region’s climate is classified, according to the Köppen criteria, as an Am monsoon climate, that is, an alternation between the rainy season and the dry season [12]. Climate data during the experimental period are shown in Figure 1. The experimental period, considering the dry period of the year, was from July 2019 to September 2019.

### 2.3. Animal Management and Experimental Diets

A total of 80 non-castrated Nelore males (testers) were used, each with a body weight of 220 ± 16.5 kg, and randomly distributed at a rate of 5 per paddock. All animals (t were weighed at the beginning of the experiment, played with, and treated against endo- and ectoparasites, and then randomly distributed among the paddocks. After 14 days of adaptation, the animals were weighed again, and at the end of the experimental period, they were fasted for 16 h from solids and liquids. In addition, regulatory animals (with the same standard as the testers) were used to maintain the herbage allowance at 7.0 kg DM 100^−1^ kg BW using the continuous stocking grazing method with a variable stocking rate (heads·ha^−1^) (put and take method). It should be noted that weight gain measurements were carried out only on the testers’ animals, which remained in all the paddocks. These adjustments allowed for the standardization of forage conditions, and the productivity variables per area (of the system) were subsequently obtained.

The treatments were distributed randomly in each paddock, with two LCs, 10 (lower) and 16.7 g·kg^−1^ (higher), and two PSs, SBM and DDGS.

The adaptation period was 14 days, starting with the supply of 2 g·kg^−1^ BW, with progressive increments to ensure that all animals ingested the same protein content per day.

The concentrates were composed of ground corn grain, livestock urea, protected urea (Optigen^®^, Alltech do Brasil, Curitiba, PR, Brasil), a mineral mixture, and a vitamin core (Table 1) and were formulated such that protein intake would be equal in all treatments (isonitrogenous) (Table 1).

### 2.4. Data Collection and Sampling Procedures

Forage collection was carried out every 28 days at ground level, and 3 samples were collected per paddock at the average height of the forage canopy, using a circular frame of known area (0.68 m²) per paddock. A subsample was taken per paddock to determine the DM, and another subsample was taken to separate the morphological components (stem, leaves, and dead material). Simultaneously, to evaluate the chemical composition of the pasture, a grazing simulation technique was carried out [13]. Forage accumulation was carried out using three exclusion cages per paddock via the paired-cage method [14]. The average height of the forage canopy was measured as described by [15]. The volumetric density of the morphological components was calculated from the total dry mass of each component divided by the average height of the pasture [16]. The calculation of grazing pressure was carried out based on the relationship between the animal’s BW (kg) and the amount of forage available (kg BW kg^−1^ DM day^−1^).

For chemical analyses of the forage and concentrates, the methods of [17] including DM, mineral matter (MM), and crude protein (CP) were used. The neutral detergent insoluble fiber (NDF) and acid detergent insoluble fiber (NDA) contents were measured according to the methods of [18], and the indigestible NDF (iNDF) content was measured according to [19]. The neutral detergent insoluble nitrogen (NDIN) and acid (ADIN) contents were determined according to [20], while the lignin content was determined according to [21], and the total digestible nutrients (TDN, g·kg^−1^ DM) were calculated according to [22]. Potentially digestible DM (pdDM) was obtained by the [23] total carbohydrate (TCHO) method according to [24].

To evaluate blood parameters, samples were collected from three animals per paddock via a puncture of the jugular vein in sterile 8 mL bottles (Vacuettes^®^, Kremsmünster, Austria) containing a coagulation accelerator, with subsequent centrifugation at 3000× *g* for 15 min, aimed at separating the plasma and serum. The serum was frozen at −20 °C for subsequent analyses of the concentrations of urea, creatinine, uric acid, alkaline phosphatase, total proteins, albumin, bilirubin, GOT/AST (aspartate aminotransferase), GPT/ALT (glutamic-pyruvic transaminase), and GAMAGT/GGT (gamma-glutamyltransferase) through an enzymatic colorimetric system using commercial kits (Gold Analisa^®^, Belo Horizonte/MG, Brazil).

### 2.5. Performance and Intake Assessment

Performance evaluations considered the weight of test animals after 16 h of fasting, whereas productivity variables were calculated considering all animals handled in each paddock. They were calculated as:

$$Average\;daily\;gain\left(ADG,kg\;BW.{day}^{-1}\right)=gain\;BW/day;$$

$$Feed\;efficiency\;\left(kg\;{kg}^{-1}\right)\;=\frac{BW\;gain\;\left(kg\right)\;}{total\;intake\;(kg)};$$

$$Stocking\;rate\;\left(AU\;{ha}^{-1},\;450\;kg\;BW.{ha}^{-1}\right)=[(\frac{\sum BWaverage}{450})/\mathrm{ha}];$$

$$Total\;weight\;gain\;per\;area\;\left(TWG.{ha}^{-1}\right)=\frac{\sum BWgain}{ha};$$

$$Gain\;weight\;per\;area\;per\;day\left(GW.{ha}^{-1}{day}^{-1}\right)=[(\frac{BWgain}{ha})/period\;in\;days];$$

$$Productivity\left(@.{ha}^{-1}\right)=\frac{TWGha}{30}$$

To estimate the actual intake of supplements (kg·day^−1^), the quantities supplied daily at 7.00 am were measured per picket, disregarding leftovers when there were any. To estimate the intake of total dry matter in the diet (total *DMI*), the following equation was used [25]:

$$Total\;DMI=-1.7824+0.07765*BW^{0.75}+4.0415*ADG-0.8973*ADG^{2}$$

where *BW* = average body weight of the animals and *ADG* = average daily gain of the animals during the period evaluated.

In addition, the disappearance of the concentrate was measured, and three assessments were carried out throughout the evaluation. At an interval of 3 h after treatment, all the concentrate present in the trough was removed, weighed, and then returned to the trough; this process was repeated until the concentrate completely disappeared or the following day.

### 2.6. Statistical Analysis

The analyses were carried out according to a completely randomized design, considering the 2 × 2 factorial arrangement (two protein sources and two levels of concentrate), totaling four treatments with four replications (paddocks) for each treatment, according to the model:

*Yklj* = *μ* + *Ak* + *Bl* + (*AB*)*kl* + *C[Yijk]* + *eklj*

where *Yklj* is the observation of the effect of the treatment; *μ* is the overall average; *Ak* is the effect of supplementation level; *Bl* is the protein source effect; *ABkl* is the effect of the interaction of the i-th level to the concentrate level with the i-th level of the protein source; and *C[Yijk]* represents the initial BW as a covariate and the *eklj* errors associated with each observation.

The individual performance results (ADGs) and serum parameters were analyzed according to the model suggested by [26] as follows:

*Ŷijk* = *μ* + *PSi* + *LCj* + *PS* × *LCij* + *Ɛijk* + *Ʊijkl*

where *Ŷijk *is the value referring to the treatment applied to the animal in paddock k in repetition j; *μ* is the overall average; *PSi* is the fixed effect of protein source, *LCj* is the fixed effect of level of concentrate j (j = 10 and 16.7 g·kg^−1^ BW), *PS* × *LC ij* is the fixed effect of the interaction between protein source and level of concentrate; *Ɛijk* is the random experimental error associated with each observation assumed to be NID~(0,σ 2); and *Ʊijkl* is the random sampling error associated with each animal (l) within the paddock (k) assumption NID~(0,σ 2). For all evaluations, the F test was used considering a 5% probability for a type I error.

## 3. Results

### 3.1. Forage Characteristics

Regardless of the level of LC or PS, forage characteristics such as height, forage accumulation, proportion, and volumetric density of leaves, stems, and dead plants did not change (Table 2; *p* > 0.050).

Interestingly, forage mass (FM) did not vary for LC (*p* = 0.330) or PS (*p* = 0.060), with an average forage availability for the period of 3506 kg DM·ha^−1^.

The level of concentrate changed the leaf/stem ratio (L/S) of the canopy (*p* = 0.030), with 21% more leaves at the lowest LC and 3.23 to 2.54 at the highest LC (Table 2).

The highest LC changed the grazing pressure (*p* > 0.050), with 82% more grazing pressure compared to the lowest LC. For the chemical composition, there was a change in the levels of crude protein and NDFap for the lowest LC (*p* < 0.050) (Table 2).

### 3.2. Animal Intake and Performance

There was an effect of LC on supplement intake (*p* < 0.050). The animals consumed 46% more in the highest LC, representing an increase of 2.04 kg in daily intake. The LC affected supplemental dry matter intake (sDMI), pasture dry matter intake (pDMI), crude protein intake (CPI), and the intake of neutral detergent insoluble fiber (INDF) (*p* < 0.050). The intake of nonfibrous carbohydrates (INFC) was affected by the LC and PS (Table 3; *p* < 0.01).

Total BW gain was affected by the LC (*p* < 0.01), representing an increase of 50.5% in the highest LC compared to the lowest LC. Similarly, the higher LC led to higher stocking rates (*p* < 0.050) and productivity (*p* < 0.010), resulting in an increase in the stocking rate of 1.36 AU·ha^−1^ and a productivity of 6.69 @·ha^−1^, respectively (Table 3).

**Figure 2:**
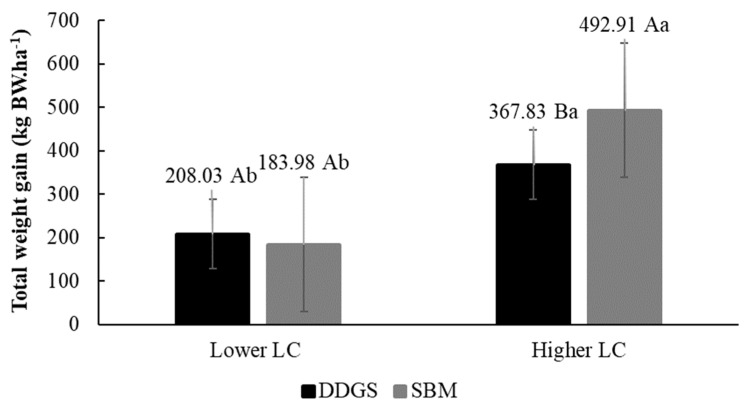
Interaction effect of LC*PS on the total weight gain (kg·ha^−1^) of beef cattle reared on pasture supplemented with high levels of concentrate and different protein sources in the dry season during the experimental period. Lower LC: Lower LC (10 g·kg^−1^ DM); Higher LC: Higher level of concentrate (16.7 g·kg^−1^ DM); DDGS: Dried Distillers Grains with Soluble; SBM: Soybean Meal. Uppercase letters indicate differences between PSs, and lowercase letters indicate differences between levels according to the F test considering a 5% probability of type I error.

For total weight gain, there was an interaction effect between the LC and PS (*p* < 0.050). The greatest increase in total weight occurred in the LC treatment with soybean meal as a source of protein, with an increase of 240.93 kg in BW·ha^−1^ with the 10 g·kg^−1^ level (Figure 2).

**Figure 3:**
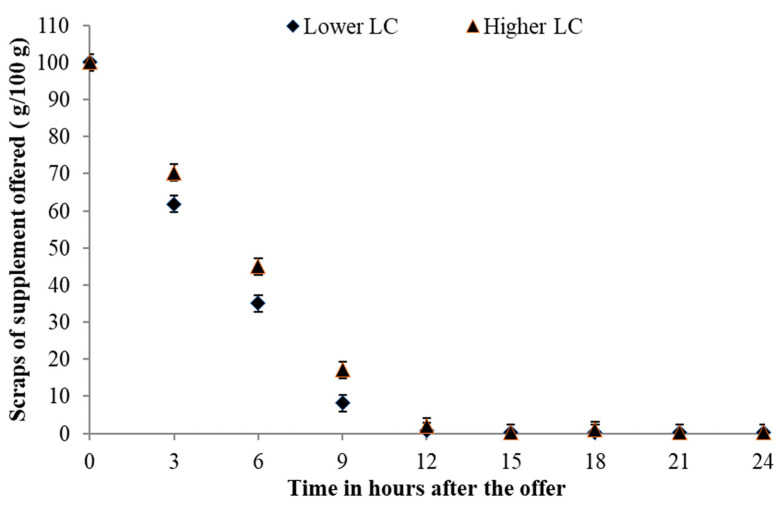
Segmented linear regression model for levels of concentrate of 10.0 (*p* < 0.0001) (lower LC) and 16.7 (higher LC) (*p* < 0.0001) g·kg^−1^ of BW for the relationship between leftovers and the time after the supplement was offered: Ŷ10.0 = 96.61 − 10.07 × Time for Time ≤ 9.53 h and Ŷ10.0 = 0.00 for Time ≥ 9.53 h (R^2^ = 99.51) and Ŷ16.7 = 99.18 − 10.07 × Time for Time ≤ 10.70 h and Ŷ16.7 = 0.00 for Time ≥ 10.70 h (R^2^ = 99.85).

**Figure 4:**
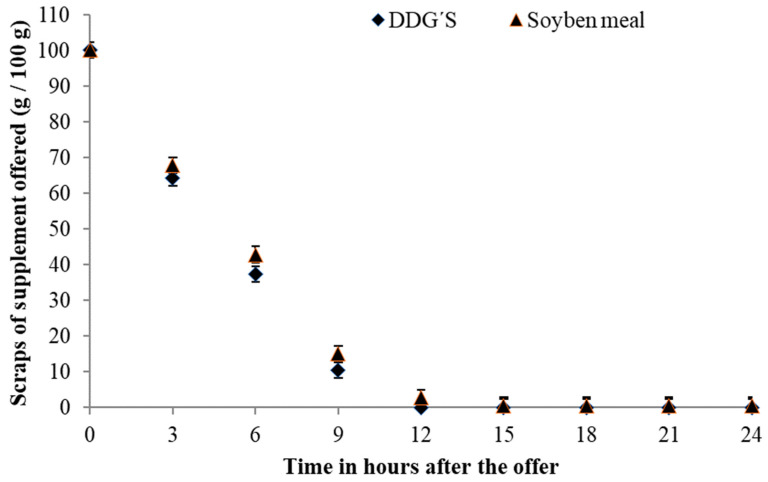
Segmented linear regression model for the protein sources DDGS (*p* < 0.0001) and SBM (*p* < 0.0001) g·kg^−1^ of BW for the relationship between leftovers and time after offering concentrate: ŶDDGS = 97.37 − 9.86 × Time for Time ≤ 9.88 h and ŶDDGS = 0.00 for Time ≥ 9.88 h (R^2^ = 99.71) and ŶSBM = 98.42 − 9.34 × Time for Time ≤ 10.31 h and ŶSBM = 0.00 for Time ≥ 10.31 h (R^2^ = 99.86).

The intake pattern regarding the disappearance of the supplement offered showed that, regardless of the LC, the animals consumed the entire supplement by noon (Figure 3; *p* > 0.050). A lower LC concentration resulted in a greater decrease, on average 9.12 g·100 g^−1^, up to 9 h after delivery. For PS (Figure 4), the intake pattern was the same, where the entire supplement was consumed by noon (*p* > 0.050).

### 3.3. Blood Parameters

There was no change in the serum profile for the variables related to PS (Table 4, *p* > 0.050).

The LC influenced (*p* = 0.001) the concentration of alkaline phosphatase, with a concentration of 86.91 U·L^−1^ for the highest LC. On the other hand, the serum urea concentration was greater, at 30.08 mg·dL^−1^, for the lowest LC (*p* = 0.043).

## 4. Discussion

This experiment was designed mainly to test the hypothesis that intensification in the rearing phase using different nutritional strategies allows for increased production in this phase during the dry period of the year. For this purpose, males of the Nelore breed in the rearing phase in the Mombaça grass pastures received two levels of concentrate and two sources of protein, and the productivity, morphological characteristics, and chemical composition of the forage were evaluated, in addition to the serum profile of the animals. The results demonstrated that the highest LC was the most interesting nutritional strategy for intensifying the rearing phase of pasture-fed beef cattle, as it guarantees greater system productivity due to the increased stocking rate. The use of DDGS as a source of protein in the supplement is an alternative to using SBM since there was no influence on any of the metrics evaluated.

The nutritional strategy is capable of influencing the morphological characteristics and forage mass, as well as the chemical composition of the forage [27]. The increase in the L/S ratio that we observed when using the lowest LC favored an increase in the CP concentration and a reduction in the NDFap of potentially grazable forage. The lower LC probably increased grazing time, possibly with a greater removal of the apical meristem by animals, reducing stem elongation, which contributed to a greater share of leaves in the forage canopy with better nutritional value [28].

Despite the difference in the L/S ratio between the LCs evaluated, this behavior was not observed for the proportions of the other morphological structures of the forage canopy (leaf, stem, and dead). It is worth noting that despite the lack of effect of LC on the proportions of the morphological structures of the Mombaça grass, a large proportion of the plants were dead or senescent (75% of dead material). As the present study took place during the dry period of the year, when forage development stops due to water stress, the green parts go through the leaf senescence process, reducing photosynthetic capacity (due to the effects of aging) [29]. The senescence process leads to the mobilization of cellular compounds from older vegetative parts, leading to their death, with the intention of ensuring the maintenance of life after experiencing severe stress [29].

Although there was no difference in total DM intake or intake to body weight for the nutritional strategies used, the highest supplement intake and the lowest pasture intake were found in the highest DM treatment group. The intake of DM of feed by cattle is an extremely important variable for determining performance [30]; throughout the day, the animals ingested 22 to 29 g·kg^−1^ BW [25]. However, intake can be affected by characteristics inherent to the food, the animal, and the interactions that may occur between them. It is known that concentrated supplementation at certain levels causes the substitution of forage intake with concentrate, leading to a lower intake of forage within the animals’ diet [31].

It was then observed that forage intake was negatively affected by the increase in LC, representing a substitutive effect of pasture intake on concentrate intake. The reduction in forage intake resulted in greater forage availability in the area, requiring more animals to maintain pasture management metrics. Low CP forages (<70 g·kg^−1^ CP) show a marked increase in protein supplement intake up to a concentrate level of 5 g·kg^−1^ CP·day^−1^ [32], which may have helped increase the rate of stocking of 1.36 AU·ha^−1^ at the highest level of concentrate with the lowest level.

The same availability of forage mass between concentrate levels helped support the hypothesis that the substitutive effect, caused by high concentrate levels, allowed different stocking rates to be obtained between treatments. Therefore, when the forage mass does not change, concentrated supplementation ensures nutritional adjustments that allow for an improvement in the individual performance of the animals, in addition to increasing productivity gains per area in pastures [33].

The nutritional strategies evaluated did not influence the final body weight of the animals, as their ADG values were similar. With the option of increasing individual performance, different nutritional strategies can be used according to the objective you wish to achieve by improving the quality of the diet ingested or those that, in addition to guaranteeing an increase in individual performance, enable an increase in productivity per area due to the increase in the stocking rate.

The ADG did not differ between the LC and PS treatments used in the evaluated strategies, demonstrating that they were able to guarantee the intake of 789.75 g of CP·day^−1^, which is necessary to achieve a gain of 1 kg of CP·day^−1^ [34]. The calculated protein intake by the animals was 0.870 and 0.980 kg of CP·day^−1^ for the lowest and highest LCs, respectively. In this way, the use of high levels of concentrate from the rearing phase of cattle on pasture favored a nutritional supply capable of guaranteeing high gains during drought, contributing to performance maintenance during the most challenging period of the year.

The greatest gain in total body weight occurred in the nutritional strategy that used the highest LC associated with SBM as a source of true protein; however, the other production variables were not influenced in the same way. A previous study [11] reported that the main source of influence on the productivity of cattle reared on pasture was the use of concentrated supplement sources in the diet, mainly favoring an increase in productivity per area. Consistent with our results, the nutritional strategy with the highest LC resulted in a yield of 6.69 @·ha^−1^, which was greater than the lowest LC. This response is mainly due to the increase in the capacity allowed by the higher LC, as the ADG differed among the factors evaluated.

The intensification of the rearing phase of cattle on pasture, in addition to allowing the benefits discussed thus far, also allows for secondary benefits such as changes in the composition of the gain [35]. An increase in the level of concentrate in grazing cattle leads to a reduction in the size of the animal’s gastrointestinal tract (GIT) to total body weight, favoring an increase in the gain yield, where animals fed diets rich in concentrate have greater carcass gains [36].

The protein composition of the supplement is a determining factor in animal performance, especially in the rearing phase. The lack of effect of PS on ADG demonstrates that the nutritional profile (protein, energy, and mineral) of DDGS guarantees its use in ruminant diets, in addition to not causing adverse effects on animal protein metabolism and nutrient intake [37]. Similarly, compared with cottonseed meal, an established protein source for soybean meal, DDGS maintained animal performance without affecting nitrogen utilization efficiency or microbial protein synthesis [38].

Furthermore, the use of DDGS has proven to be an interesting alternative for feeding ruminants in different production systems, as high concentrations of DDGS are used in concentrated supplements without compromising individual performance or increasing the risk of metabolic disorders [38].

When DDGS was used in a finishing diet, maintenance performance and carcass and meat parameters were observed compared to those of traditional protein sources [38]. The main point for the use of DDGS in animal feed is the cost and its availability in the region, as there are already several studies that guarantee its quality in ruminant feed [38].

The disappearance of supplements in the different nutritional strategies had a similar behavior, where almost all the supplements had been consumed within 12 h of supply. As it is a high-intake supplement with a high inclusion of corn, the conventional techniques used in formulations to regulate intake (inclusion of sodium and urea) would be inefficient, with only the physiological control acting on supplement intake.

The highest intake was observed in the first three hours after feeding, as [39] the animals choose to graze at milder temperatures throughout the day, which could be in the morning or late afternoon [39]. However, the low availability and inferior quality of forage observed meant that animals spent more time grazing to intake all the forage they needed, where grazing time is dependent on the availability and quality of forage [40]. In this way, in the first hours after treatment, the animals chose to ingest this because it was the main source of food and easier access, and during the remainder of the time, they searched the pasture for the remainder necessary to meet their demand, which had not yet been processed and/or attended to.

The concentrations of total protein, albumin, and urea in blood serum are indicators of the adequacy or inadequacy of nitrogen in the animal diet [41]. Urea is a short-term blood parameter, while albumin is a long-term response [42]. In the present study, the albumin and total protein levels were lower than those reported by [41] for Brahman breed steers fed with two levels of concentrate (5 and 15 g·kg^−1^ BW) but within the Nellore breed standard (30.8–35.8 g.L^−1^) [43].

Values for blood urea within the ideal range (<64.86 mg·dL^−1^) in cattle indicate that the effective rumen degradable protein (RDP) is adequate [44]. High blood urea levels may indicate high protein intake or excessive muscle mobilization [45]. Previous research suggested that blood urea concentrations greater than approximately 5 to 9 mg·dL^−1^ indicate excessive N intake and N wastage [46,47]. At all inclusion levels of LC used, the blood urea levels were above 9 mg·dL^−1^ (Table 4), suggesting that the CP concentration of the diets used may be above the minimum concentration necessary for the animals. This would correspond to levels of ruminal ammonia nitrogen above those that could be used by ruminal microorganisms [48].

## 5. Conclusions

The use of a higher LC is a more interesting nutritional strategy to intensify the rearing phase of beef cattle on pasture during the dry season to guarantee greater productivity of the system due to the increased stocking rate, increased weight gain per area, and maintenance of animal serum standards within the safe range.

Furthermore, the use of DDGS as a PS in the supplementation of the rearing phase of pasture-fed beef cattle during the dry season is an alternative to using SBM, since there was no change in any of the metrics evaluated.

## Figures and Tables

**Table 1:** Proportion of ingredients and chemical composition of nutritional strategies for intensive rearing of beef cattle.

Items	Supplements				Pasture (Mombasa Grass)
	Higher/SBM ^15^	Higher/DDGS ^16^	Lower/SBM ^17^	Lower/DDGS ^2^	
**Proportion of ingredients ^1^**					
Ground corn grain	809.10	729.41	538.55	320.92	-
Soybean meal	135.44	-	374.52	-	-
DDGS ^2^	-	215.62	-	594.25	-
Livestock Urea	13.38	13.35	22.10	21.99	-
Protected urea	6.69	6.68	11.05	11.00	-
Common Salt	5.96	6.09	8.84	8.80	-
Limestone	11.98	15.24	23.15	32.09	-
Foscalcium	13.31	8.59	15.04	2.04	-
BYPRO ^3^	1.46	1.45	2.45	2.44	-
PREMIX MIN. VIT ^4^	2.70	3.57	4.30	6.47	-
**Chemical composition ^1^**					
DM ^5^	898.50	904.00	908.40	907.00	580.60
pdDM ^6^	967.30	969.70	946.30	944.70	673.76
MM ^7^	59.17	60.87	84.07	84.08	90.75
CP ^8^	193.93	211.05	311.14	299.65	32.17
EE ^9^	32.83	37.99	20.64	48.63	12.70
NDFap ^10^	62.2	109.4	90.4	211.9	781.01
ADF ^11^	69.87	95.58	75.70	145.06	425.76
iNDF ^12^	14.72	13.17	36.26	40.09	309.53
NFC ^13^	686.59	615.32	551.03	412.73	202.06
TDN ^14^	836.13	804.38	804.35	740.48	551.04

^1^ g·kg^−1^ DM; ^2^ Dried Distillers Grains with Solubles (DDGS); ^3^ Food supplement containing 70% condensed tannins extracted from Quebracho-vermelho (*Schinopsis lorentzii*); ^4^ Fortuna Animal Nutrition: Vitamitract ADE, Fe Sulfate 30%, Mn Sulfate 26%, Chromium Chelate 10%, Sulfur Vent 99%. ^5^ Dry matter; ^6^ Potentially digestible dry matter; ^7^ Mineral matter; ^8^ Crude protein; ^9^ Ethereal extract; ^10^ Neutral detergent fiber corrected for ash and protein; ^11^ Acid detergent fiber; ^12^ Indigestible fiber in neutral detergent; ^13^ Nonfibrous carbohydrates; ^14^ Total digestible nutrients (calculated by the NRC formula, 2021); ^15^ Soybean meal; ^16^ Higher level of concentrate; ^17^ Lower level of concentrate.

**Table 2:** Characteristics of *Megathyrsus maximus* cv. Mombaça grass under continuous stocking grazed by beef cattle supplemented with higher and lower levels of concentrate and different protein sources, Dried Distillers grains with Solubles (DDGS) and Soybean Meal (SBM), during the dry period of the year.

Items	LC ^9^		PS ^10^		SEM ^1^	*p* Value		
	Lower	Higher	DDGS	SBM		LC	PS	LC × PS
Height (cm)	39.5	37.4	38.9	37.9	1.07	0.21	0.53	0.86
FM ² (kg DM·ha^−1^)	3648	3364	3797	3214	194.9	0.33	0.06	0.47
Leaf proportion (g·100·g^−1^)	18.1	17.4	16.2	19.3	1.63	0.77	0.20	0.97
Stem proportion (g·100·g^−1^)	6.42	6.67	6.34	6.74	0.37	0.55	0.36	0.75
Dead proportion (g·100·g^−1^)	74.1	75.8	76.0	73.9	1.71	0.36	0.29	0.47
L: S ³	3.23 a	2.54 b	2.89	2.88	0.19	0.03	0.96	0.3
VD ^4^ leaf (kg·DM·ha^−1^ cm)	13.4	12.2	13.5	12.1	0.89	0.28	0.23	0.46
VD ^4^ stem (kg·DM·ha^−1^ cm)	4.38	4.31	4.39	4.30	0.25	0.85	0.81	0.31
VD ^4^ total (kg·DM·ha^−1^ cm)	95.6	92.0	99.9	87.7	4.93	0.58	0.08	0.43
FA ^5^ (kg DM·ha·day^−1^)	55.7	55.6	58.7	52.6	5.43	0.99	0.30	0.50
Grazing pressure	0.17 b	0.31 a	0.22	0.26	0.02	< 0.01	0.12	0.13
**Chemical composition**								
Dry matter (g·100·g^−1^)	57.5	58.7	57.7	58.4	0.57	0.07	0.26	0.28
pdDry matter ^6^ (g·100·g^−1^)	69.2	68.3	69.4	68.2	6.24	0.34	0.22	0.77
Organic matter (g·kg^−1^)	909.2	909.3	910.7	907.7	1.42	0.96	0.17	0.21
Crude protein (g·kg^−1^)	34.2 a	30.1 b	31.9	32.4	0.86	0.01	0.66	0.36
NDFap ^7^ (g·kg^−1^)	712.9 b	733.4 a	720.5	725.7	2.52	0.01	0.18	0.13
iNDF ^8^ (g·kg^−1^)	300.3	310.6	299.2	311.7	4811	0.16	0.10	0.56

^1^ standard error of the mean. ^2^ Forage mass; ^3^ Leaf/stem ratio; ^4^ Volumetric density; ^5^ Forage accumulation; ^6^ Potentially digestible dry matter; ^7^ Neutral detergent fiber corrected for ash and protein; ^8^ Indigestible neutral detergent fiber; ^9^ Level of Concentrate: Lower (10 g kg^−1^ DM) and Higher (16.7 g kg^−1^ DM); ^10^ Protein sources. Means followed by the same letters on the line do not differ from each other according to the F test considering a 5% probability of type I error.

**Table 3:** The performance of beef cattle reared on *Megathyrsus maximus* pasture cv. Mombaça grass supplemented with higher and lower levels of concentrate and different protein sources, Dried Distillers grains with Solubles (DDGS) and Soybean Meal (SBM), during the dry period of the year.

Items	LC ^10^		PS ^11^		SEM ¹	*p* Value		
	Lower	Higher	DDGS	SBM		LC	PS	LC × PS
Initial body weight (kg)	242	242	242	243	3.04	0.95	0.79	0.52
Final body weight (kg)	319	324	322	321	4.24	0.33	0.94	0.32
Concentrate intake (kg·DM·day^−1^)	2.42 b	4.46 a	3.48	3.40	0.12	<0.01	0.47	0.42
Supplement intake (% BW·day^−1^)	0.77 b	1.38 a	1.08	1.07	0.04	<0.01	0.74	0.94
Average daily gain (kg·day^−1^·animal^−1^)	0.92	1.02	0.97	0.97	0.04	0.11	0.98	0.15
Feed efficiency (g·kg^−1^)	14.3	14.7	14.6	14.5	0.47	0.37	0.90	0.29
Total body weight gain (kg·BW·ha^−1^)	196 b	396 a	288	304	45.7	<0.01	0.35	0.04
Body weight gain area (kg·BW·day·ha^−1^)	2.41 b	4.87 a	3.47	3.81	0.22	<0.01	0.19	0.09
Stocking rate (AU·ha^−1^)	1.78 b	3.14 a	2.30	2.63	0.17	0.01	0.20	0.54
Productivity (@·ha^−1^)	6.54 b	13.2 a	9.43	10.3	0.59	<0.01	0.18	0.09
**Nutrient intake ^2^**								
Total DMI ^3^ (kg·DM·day^−1^)	6.51	6.66	6.59	6.59	0.14	0.36	0.99	0.53
DMI %BW ^4^	2.33	2.35	2.34	2.33	0.13	0.61	0.86	0.37
sDMI ^5^ (kg·DM·day^−1^)	2.38 b	4.46 a	3.40	3.44	0.16	<0.01	0.23	0.06
pDMI ^6^ (kg·DM·day^−1^)	4.13 a	2.27 b	3.14	3.26	0.17	<0.01	0.50	0.49
CPI ^7^ (kg·DM·day^−1^)	0.87 b	0.98 a	0.93	0.91	0.01	<0.01	0.16	0.44
INDF ^8^ (kg·DM·day^−1^)	3.39 a	2.16 b	2.86	2.68	0.09	<0.01	0.19	0.66
INFC ^9^ (kg·DM·day^−1^)	1.98 b	3.35 a	2.51 b	2.82 a	0.03	<0.01	<0.01	0.38

^1^ Standard error of the mean; ^2^ Intake was calculated according to equation 2.1, BR Cut 3.0; ^3^ Total dry matter intake; ^4^ Dry matter intake in % of body weight; ^5^ Supplement dry matter intake; ^6^ Pasture dry matter intake; ^7^ Crude protein intake; ^8^ Intake of neutral detergent fiber; ^9^ Intake of nonfibrous carbohydrates; ^10^ Level Concentrate: Lower (10 g kg^−1^ DM) and Higher (16.7 g·kg^−1^ DM); ^11^ Protein sources. Means followed by the same letters on the line do not differ from each other according to the F test considering a 5% probability of type I error.

**Table 4:** Serum profile of beef cattle receiving different nutritional strategies as a strategy to intensify the rearing phase in the dry period of the year.

Items	LC ^5^		PS ^6^		SEM ¹	*p* Value		
	Lower	Higher	DDGS	SBM		LC	PS	LC × PS
Cholesterol (mg·dL^−1^)	95.8	95.7	102.3	89.1	6.16	0.99	0.11	0.52
Uric Acid (mg·dL^−1^)	4.5	4.72	4.76	4.46	0.48	0.75	0.66	0.35
Albumin (g·L^−1^)	33.9	33.6	34.1	35.3	1.21	0.32	0.50	0.17
Total Proteins (g·L^−1^)	84.5	79.9	79.6	84.8	4.12	0.43	0.38	0.28
GOT ^2^ (U·L^−1^)	84.9	76.3	77.8	83.4	5.48	0.27	0.46	0.46
GTP ^3^ (U·L^−1^)	74.4	83.6	79.5	78.5	5.32	0.22	0.89	0.98
Alkaline Phosphatase (U·L^−1^)	60.2 b	86.9 a	70.1	77.0	5.29	<0.01	0.35	0.23
Urea (mg·dL^−1^)	30.1 a	25.1 b	27.0	28.1	1.57	0.04	0.06	0.18
Gamma GT ^4^ (U·L^−1^)	45.2	36.3	43.9	37.6	6.45	0.32	0.49	0.06

¹ Standard error of the mean; ^2^ Glutamic oxalacetic transaminases; ^3^ Glutamic Transaminase–pyruvic; ^4^ Gamma glutamyl transferase; ^5^ Levels Concentrate: Lower (10 g·kg^−1^ DM) and Higher (16.7 g·kg^−1^ DM); ^6^ Protein source: Dried Distillers grains With Solubles (DDGS) and Soybean Meal (SBM). Means followed by identical letters on the line do not differ from each other according to the F test considering a 5% probability of type I error.

## Data Availability

The data presented in this study are available upon request from the corresponding author.
